# Synthesis of Some New Pyridine-2,6-carboxamide-derived Schiff Bases as Potential Antimicrobial Agents

**DOI:** 10.3390/molecules15074711

**Published:** 2010-07-06

**Authors:** Mohamed A. Al-Omar, Abd El-Galil E. Amr

**Affiliations:** Department of Pharmaceutical Chemistry, College of Pharmacy, King Saud University, Riyadh 11451, Saudi Arabia

**Keywords:** 2,6-pyridinedicarbonyl dichloride, pyridine-2,6-carboxamides, Schiff's base, antimicrobial activity

## Abstract

A series of pyridine-bridged 2,6-bis-carboxamide Schiff's bases has been prepared starting from 2,6-pyridinedicarbonyl dichloride (**1**) and L-alanine or 2-methyl-alanine methyl ester. The coupling of acid chloride **1 **with L-alanine methyl ester hydrochloride -or 2-methylalanine methyl ester hydrochloride gave the corresponding 2,6-bis-carboxamide pyridine methyl esters **2a,b**. Hydrazonolysis of **2** with hydrazine hydrate afforded the corresponding bis-hydrazides **3a,b**. Treatment of **3a,b **with appropriate aromatic or heterocyclic aldehydes afforded the corresponding pyridine- bridged 2,6-bis-carboxamide Schiff's bases **4a-f** and **5a-f**, respectively. The newly synthesized compounds **2-5** were screened for their bactericidal and fungicidal activities. Many of the obtained compounds exhibited significant antimicrobial activity, comparable to streptomycin and fusidic acid, which were used as reference antibiotic drugs.

## 1. Introduction

In our previous work, we have reported that certain of substituted pyridine and Schiff base derivatives as antimicrobial, anti-inflammatory and anticancer agents [1,2,3,4,5,6]. Also, Schiff base and other heterocyclic derivatives were reported to possess diverse biological activities, such as antibacterial [7,8,9,10] and anti-inflammatory [11,12,13] properties. In addition, several substituted pyridines and their derivatives were reported to exhibit significant antimicrobial [14], anti-inflammatory [15] and anticancer activities [16]. In continuation of our interest in the chemical and pharmacological properties of disubstituted pyridine derivatives [17,18,19,20], we report herein the synthesis and antimicrobial activities of a new series of hydrazides and their corresponding N^2^,N^6^-bis(1-oxo-1-(2-(substituted-benzylidene)-hydrazinyl)propan-2-yl)pyridine-2,6-di-carboxamide derivatives (Schiff's bases). 

## 2. Results and Discussion

### 2.1. Chemistry

**Scheme 1:**
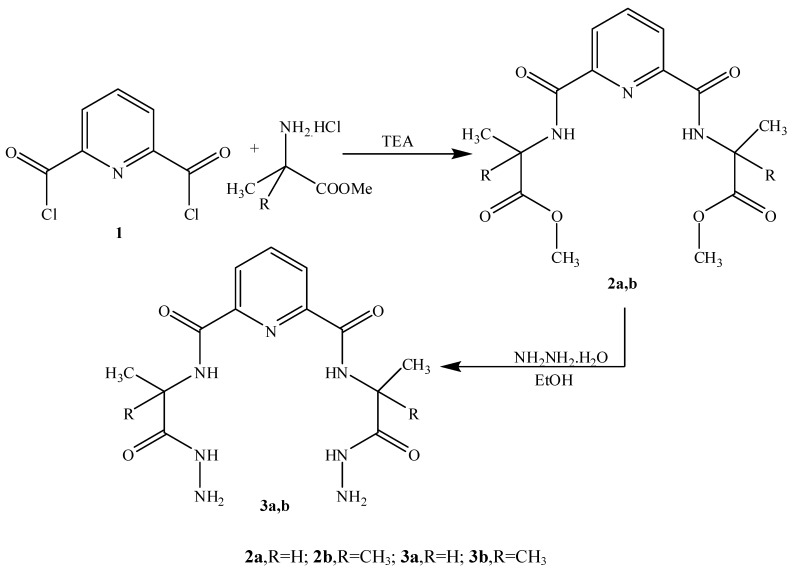
Synthetic Pathway for Compounds **2a,b **and **3a,b**.

L-Alanine and/or 2-methylalanine methyl esters were initially coupled with 2,6-pyridinedicarbonyl dichloride (**1**) (acid chloride method) [21] to give the corresponding 2,6-bis-carboxamide pyridine methyl esters **2a,b**. Treatment of 2,6-bis-esters **2a,b** with hydrazine hydrate in absolute ethanol afforded the corresponding 2,6-bis-hydrazides **3a,b** (Scheme 1). Some physical properties of these compounds are listed in Table 1.

**Table 1:** Melting points, crystallization solvents, yields, molecular formulae and molecular weights of compounds **2a,b **and **3a,b**.

Comp. No.	R	Mp (ºC)	Cryst. Solv.	Yield (%)	[α]^30^_D_	Molecular Formula (Mol. Wt.)
**2a**	H	182-184	EtOH	75	+15 (DMF)	C_15_H_19_N_3_O_6_ (337.33)
**2b**	CH_3_	196-198	EtOH	68	-	C_17_H_23_N_3_O_6_ (365.38)
**3a**	H	252-254	AcOH/H_2_O	82	+56 (DMF)	C_13_H_19_N_7_O_4_ (337.33)
**3b**	CH_3_	246-248	AcOH/H_2_O	85	-	C_15_H_23_N_7_O_4_ (365.39)

**Scheme 2:**
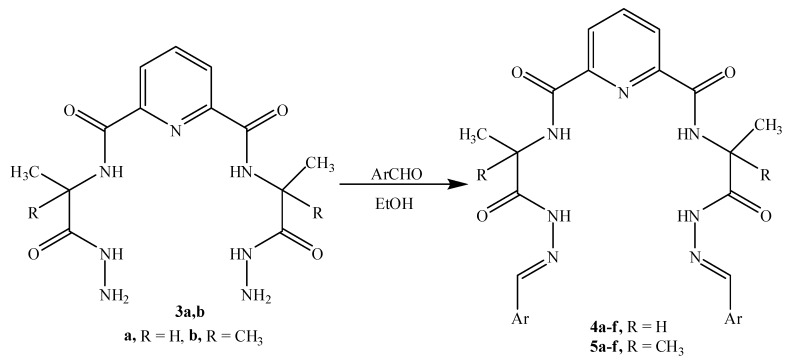
Synthetic Pathway for Compounds **4a-f **and **5a-f**.

Compounds **4a-f **and **5a-f** are new, and were synthesized via simple condensation of the hydrazides **3a,b** with appropriate aromatic or heterocyclic aldehydes, namely, benzaldehyde, *p*-methoxy-benzaldehyde, 3,4,5-trimethoxybenzaldehyde, *p*-chlorobenzaldehyde, 2-chloro-6-flourobenzaldehyde, and/or 2-thiophenealdehyde in refluxing absolute ethanol giving the corresponding N^2^,N^6^-bis(1-(2-(substituted benzylidene)hydrazinyl)-1-oxopropan-2-yl)pyridine-2,6-dicarboxamides **4a-f **and N^2^,N^6^-bis(1-(2-(substituted benzylidene)hydrazinyl)-2-methyl-1-oxopropan-2-yl)pyridine-2,6-dicarboxamides **5a-f** (Scheme 2)**.**

**Table 2:** Melting points, crystallization solvents, yields, specific rotation, molecular formulae and molecular weights of compounds **4a-h **and **5a-h**.

Comp. No.	Ar	Mp (ºC)	Cryst. Solv.	Yield (%)	[α]^30^_D_ (DMF)	Molecular Formula (Mol. Wt.)
**4a**	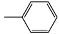	122-124	EtOH/ *n*-hexane	68	+ 18	C_27_H_27_N_7_O_4_(513.55)
**4b**	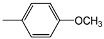	210-212	AcOH/H_2_O	75	+ 32	C_29_H_31_N_7_O_6_(573.60)
**4c**	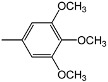	148-150	AcOH	80	+ 24	C_33_H_39_N_7_O_10_ (693.70)
**4d**	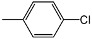	205-207	EtOH/ *n*-hexane	65	+ 54	C_27_H_25_Cl_2_N_7_O_4_ (582.44)
**4e**	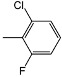	168-170	AcOH/H_2_O	72	+ 12	C_27_H_23_Cl_2_F_2_N_7_O_4_ (618.42)
**4f**	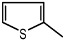	185-187	EtOH/ *n*-hexane	60	+ 16	C_23_H_23_N_7_O_4_S_2_ (525.60)
**5a**	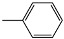	240-242	EtOH	70	-	C_29_H_31_N_7_O_4_ (541.60)
**5b**	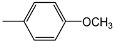	120-122	Dioxane	75	-	C_31_H_35_N_7_O_6_ (601.65)
**5c**	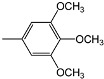	135-137	AcOH/H_2_O	66	-	C_35_H_43_N_7_O_10_ (721.76)
**5d**	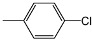	155-157	AcOH/H_2_O	78	-	C_29_H_29_Cl_2_N_7_O_4_ (610.49)
**5e**	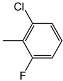	213-215	AcOH/H_2_O	86	-	C_29_H_27_Cl_2_F_2_N_7_O_4_ (646.47)
**5f**	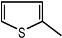	220-222	EtOH	60	-	C_25_H_27_N_7_O_4_S_2_ (553.66)

The structures of all the newly synthesized compounds **2a,b, 3a,b, 4a-f **and **5a-f** were confirmed by their IR, ^1^H-NMR, ^13^C-NMR and mass spectra.

### 2.2. Antimicrobial testing

Preliminary biological activity screening of the synthesized compounds has been performed at 50 μg/mL against microorganisms representing Gram-positive bacteria (*Bacillus subtilis* and *Staphylococcus aureus*), Gram-negative bacteria (*Escherichia coli*) and fungi (*Candida albicans* and *Aspergillus niger*), using the bioassay technique for antibiotics [22] specified in the US Pharmacopeia. From Table 3 it appears that the Schiff's bases **4b-f** and **5b-f** have significant antimicrobial activities. Among these Schiff's bases, the 4-methoxy- **4b,5b**, 3,4,5-trimethoxy- **4c,5c**, 4-chloro-**4d,5d**, 2-chloro-6-flouro-**4e,5e** and 2-thienyl- derivatives **4f,5f** have antimicrobial activities higher those of **4a,5a** with an unsubstituted phenyl group. The hydrazides **3a,b** were found to have lower antimicrobial activities, while the esters **2a,b** didn’t show any antifungal activity. Streptomycin and fusidic acid were used as antibacterial and antifungal reference drugs, respectively.

**Table 3:** Antimicrobial activities of the new synthesized compounds **2a,b, 3a,b, 4a-f **and **5a-f**.

Comp. No.	Inhibition zones (cm)				
	Gram-positive		Gram-negative	Fungi	
	*Bacillus subtilis*	*Staphylococcus aureus*	*Escherichia* * coli*	*Candida albicans*	*Aspergillus niger*
**2a**	15	18	16	-	-
**2b**	14	13	15	-	-
**3a**	1	12	13	14	10
**3b**	12	14	15	12	11
**4a**	14	15	16	14	12
**4b**	20	19	19	16	15
**4c**	21	18	20	17	16
**4d**	20	19	21	17	14
**4e**	22	20	20	18	16
**4f**	21	18	18	17	16
**5a**	16	15	17	12	10
**5b**	21	18	20	14	15
**5c**	19	20	19	16	16
**5d**	20	17	18	16	14
**5e**	22	20	20	17	14
**5f**	20	19	21	16	15
**Streptomycin**	22	21	22	-	-
**Fusidic acid**	-	-	-	18	17

## 3. Experimental

### 3.1. General

Melting points (ºC) were measured in open glass capillaries using a Barnstead 9001 Electrothermal melting point apparatus and are uncorrected. NMR spectra were obtained on a Bruker AC 500 Ultra Shield NMR spectrometer (Bruker, Fällanden, Switzerland) operating at 500.13 MHz for ^1^H and 125.76 MHz for ^13^C; the chemical shifts are expressed in δ (ppm) downfield from tetramethylsilane (TMS) used as internal standard. Electrospray ionization mass spectra (ESI-MS) were recorded on a Waters QuatroMicro triple quadrupole tandem mass spectrometer at 4.0 and 3.5 kV for positive and negative ions, respectively. Elemental analyses (C, H, N, Cl, S) were in full agreement with the proposed structures within ± 0.4% of the theoretical values. Monitoring of reactions and checking of purity of the final products were carried out by thin layer chromatography (TLC) using silica gel precoated aluminum sheets (60 F254, Merck) and visualization with ultraviolet light (UV) at 365 and 254 nm.

### 3.2. Chemistry

#### 3.2.1. *N^2^,N^6^-Bis(1-methoxy-oxopropan-2-yl)pyridine-2,6-dicarboxamides* **2a,b**

To a solution of L-alanine and/or 2-methylalanine methyl esters (2 mmol), 2,6-pyridinedicarboyl dichloride **1** (0.204 g, 1 mmol) in dichloromethane (15 mL) was added at -10 ºC with stirring. Triethylamine was added dropwise to the reaction mixture in order to keep the reaction mixture slightly basic (pH ~ 8). Stirring was continued for 3 h more at -15 ºC and then 12 h at r.t. The reaction mixture was then washed with water, 1N hydrochloric acid, 1N sodium bicarbonate and finally with water and dried over anhydrous calcium chloride. The solvent was evaporated under reduced pressure to dryness and the obtained solid was crystallized from the appropriate solvent indicated in Table 1 to give the corresponding bis-esters **2a,b**.

*N^2^,N^6^-Bis(1-methoxy-1-oxopropan-2-yl)pyridine-2,6-dicarboxamide* (**2a**). IR (KBr, cm^-1^): ν 3352-3268 (NH), 1745 (C=O, ester), 1678 (C=O, amide); ^1^H-NMR (DMSO-d_6_): δ 1.32 (d, 6H, 2 CH_3_), 3.62 (s, 6H, 2OCH_3_), 4.25 (m, 2H, 2CH), 8.18-8.26 (m, 3H, pyridine-H), 8.62 (s, 2H, 2NH exchangeable with D_2_O); ^13^C-NMR: 17.32 (2C, 2CH_3_), 47.88 (2C, 2CH), 56.15 (2C, 2OCH_3_), 123.12, 138.54, 149.65 (5C, pyridine-C), 159.96, 171.86 (4C, 4C=O); MS, *m/z* (%): 337 (M^+^, 5), 306 (15), 275 (100), 219 (12), 163 (26), 133 (75), 105 (14), 77 (65).

*N^2^,N^6^-Bis(1-methoxy-2-methyl-1-oxopropan-2-yl)pyridine-2,6-dicarboxamide* (**2b**). IR (KBr, cm^-1^): ν 3332-3278 (NH), 1747 (C=O, ester), 1676 (C=O, amide); ^1^H-NMR (DMSO-d_6_): δ 1.46 (s, 12H, 4 CH_3_), 3.56 (s, 6H, 2OCH_3_), 8.15-8.28 (m, 3H, pyridine-H), 8.42 (s, 2H, 2NH exchangeable with D_2_O); ^13^C-NMR: 23.78 (4C, 4CH_3_), 55.18 (2C, NH-C(CH_3_)_2_CO), 123.18, 139.05, 149.72 (5C, pyr-C), 160.12, 172.65 (4C, 4C=O); MS, *m/z* (%): 365 (M^+^, 8), 334 (25), 303 (80), 218 (100), 148 (6), 133 (42), 105 (34), 77 (78).

#### 3.2.2. * N^2^,N^6^-Bis(1-hydrazinyl)pyridine-2,6-dicarboxamides* **3a,b**

A mixture of bis-esters **2a** or **2b** (1 mmol) and hydrazine hydrate (0.8 mL, 16 mmol) in absolute ethanol (50 mL) was refluxed for 6 h. Excess solvent was evaporated under reduced pressure to dryness, the obtained residue was triturated with ethanol and the resulting solid was crystallized from the appropriate solvent to give bis-hydrazide derivatives **3a,b**, respectively (Table 1).

*N^2^,N^6^-Bis(1-hydrazinyl-1-oxopropan-2-yl)pyridine-2,6-dicarboxamide* (**3a**). IR (KBr, cm^-1^): ν 3465-3228 (NH, NH_2_), 1680, 1675 (2C=O, amide); ^1^H-NMR (DMSO-d_6_): δ 1.36 (d, 6H, 2 CH_3_), 4.12 (s, 4H, 2NH_2_ exchangeable with D_2_O), 4.62 (m, 2H, 2CH), 8.12-8.24 (m, 3H, pyridine-H), 8.68, 9.05 (2s, 4H, 4NH exchangeable with D_2_O); ^13^C-NMR: 18.05 (2C, 2CH_3_), 50.12 (2C, 2CH), 123.34, 139.05, 149.78 (5C, pyridine-C), 160.08, 171.24 (4C, 4C=O); MS, *m/z* (%): 337 (M^+^, 15), 321 (8), 305 (5), 275 (10), 219 (12), 176 (16), 133 (100), 105 (24), 77 (45).

*N^2^,N^6^-Bis(1-hydrazinyl-2-methyl-1-oxopropan-2-yl)pyridine-2,6-dicarboxamide* (**3b**). IR (KBr, cm^-1^): ν 3470-3218 (NH, NH_2_), 1678, 1672 (2C=O, amide); ^1^H-NMR (DMSO-d_6_): δ 1.35 (s, 12H, 4 CH_3_), 4.15 (s, 4H, 2NH_2_ exchangeable with D_2_O), 8.18-8.26 (m, 3H, pyridine-H), 8.18, 8.98 (2s, 4H, 4NH exchangeable with D_2_O); ^13^C-NMR: 25.86 (4C, 4CH_3_), 59.64 (2C, NH-C(CH_3_)_2_CO), 123.42, 139.00, 149.88 (5C, pyridine-C), 160.08, 178.65 (4C, 4C=O); MS, *m/z* (%): 365 (M^+^, 4), 333 (5), 233 (100), 148 (65), 133 (48), 105 (56), 77 (76).

#### 3.2.3. General procedure for the synthesis of *N^2^,N^6^-bis(1-(substituted)pyridine-2,6-dicarboxamides* **4a-f** *and* **5a-f**

A mixture of the hydrazide derivative **3a** or **3b** (1 mmol) and the appropriate aldehyde, namely benzaldehyde, *p*-methoxybenzaldehyde, 3,4,5-trimethoxybenzaldehyde, *p*-chlorobenzaldehyde, 2 chloro-6-flourobenzaldehyde, and/or 2-thiophenealdehyde (2 mmol) in absolute ethanol (25 mL) was heated under reflux for 4-6 h. The excess solvent was evaporated under reduced pressure, the residue was washed with *n*-hexane and triturated with diethyl ether. The obtained solid was filtered off, washed with ether, and crystallized from the appropriate solvent (see Table 2) to give the corresponding dicarboxamide derivatives **4a-f** and **5a-f**, respectively.

*N^2^,N^6^-Bis(1-(2-benzylidenehydrazinyl)-1-oxopropan-2-yl)pyridine-2,6-dicarboxamide* (**4a**). IR (KBr, cm^‑1^): ν 3356-3198 (NH), 1675, 1674 (2C=O, amide); ^1^H-NMR (DMSO-d_6_): δ 1.32 (d, 6H, 2 CH_3_), 4.56 (m, 2H, 2CH), 7.15-7.76 (m, 10H, 2Ph-H), 8.16-8.35 (m, 5H, pyridine-H + 2CH=N), 8.72, 10.54 (2s, 4H, 4NH exchangeable with D_2_O); ^13^C-NMR: 17.76 (2C, 2CH_3_), 50.85 (2C, 2CH), 127.16, 128.60, 130.48, 132.98 (12C, 2Ph), 123.36, 138.70, 149.56 (5C, pyridine-C), 143.75 (2C, 2 CH=N), 160.28, 176.86 (4C, 4C=O); MS, *m/z* (%): 514 (M^+^+1, 12), 436 (4), 359 (16), 317 (24), 275 (100), 245 (65), 216 (32), 176 (46), 133 (90), 105 (64), 77 (52).

*N^2^,N^6^-Bis(1-(2-(4-methoxybenzylidene)hydrazinyl)-1-oxopropan-2-yl)pyridine-2,6-dicarboxamide* (**4b**). IR (KBr, cm^-1^): ν 3348-3210 (NH), 1680, 1676 (2C=O, amide); ^1^H-NMR (DMSO-d_6_): δ 1.34 (d, 6H, 2 CH_3_), 3.72 (s, 6H, 2OCH_3_), 4.52 (m, 2H, 2CH), 7.18 (d, 4H, Ar-H), 7.78 (d, 4H, Ar-H), 8.18 (s, 2H, 2CH=N), 8.23-8.32 (m, 3H, pyridine-H), 8.68, 10.62 (2s, 4H, 4NH exchangeable with D_2_O); ^13^C-NMR: 17.72 (2C, 2CH_3_), 51.05 (2C, 2CH), 54.66 (2C, 2OCH_3_), 114.05, 125.60, 129.78, 162.56 (12C, 2Ar-C), 123.30, 138.75, 149.50 (5C, pyridine-C), 144.00 (2C, 2 CH=N), 161.02, 176.82 (4C, 4C=O); MS, *m/z* (%): 574 (M^+^+1, 6), 542 (12), 511 (6), 353 (24), 322 (18), 220 (100), 204 (65), 189 (13), 133 (76), 118 (46), 105 (46), 77 (44). 

*N^2^,N^6^-Bis(1-oxo-1-(2-(3,4,5-trimethoxybenzylidene)hydrazinyl)propan-2-yl)pyridine-2,6-dicarbox-amide* (**4c)**. IR (KBr, cm^-1^): ν 3354-3218 (NH), 1677, 1674 (2C=O, amide); ^1^H-NMR (DMSO-d_6_): δ 1.28 (d, 6H, 2 CH_3_), 3.76 (s, 18H, 6OCH_3_), 4.48 (m, 2H, 2CH), 7.12 (s, 4H, Ar-H), 8.14 (s, 2H, 2CH=N), 8.24-8.36 (m, 3H, pyridine-H), 8.72, 10.76 (2s, 4H, 4NH exchangeable with D_2_O); ^13^C-NMR: 18.05 (2C, 2CH_3_), 51.55 (2C, 2CH), 55.10 (4C, 4OCH_3_), 58.72 (2C, 2OCH_3_), 104.52, 127.65, 140.68, 152.82 (12C, 2Ar-C), 123.45, 139.06, 149.65 (5C, pyridine-C), 145.18 (2C, 2 CH=N), 161.25, 176.88 (4C, 4C=O); MS, *m/z* (%): 694 (M^+^+1, 4), 662 (8), 631 (12), 526 (18), 412 (28), 280 (100), 204 (45), 189 (8), 167 (34), 133 (82), 105 (66), 77 (56).

*N^2^,N^6^-Bis(1-(2-(4-chlorobenzylidene)hydrazinyl)-1-oxopropan-2-yl)pyridine-2,6-dicarboxamide* (**4d**). IR (KBr, cm^-1^): ν 3352-3198 (NH), 1677, 1675 (2C=O, amide); ^1^H-NMR (DMSO-d_6_): δ 1.28 (d, 6H, 2 CH_3_), 4.48 (m, 2H, 2CH), 7.42 (d, 4H, Ar-H), 7.65 (d, 4H, Ar-H), 8.16-8.35 (m, 5H, pyridine-H + 2CH=N), 8.72, 10.82 (2s, 4H, 4NH exchangeable with D_2_O); ^13^C-NMR: 18.04 (2C, 2CH_3_), 50.95 (2C, 2CH), 127.82, 128.62, 130.88, 135.76 (12C, 2Ar-C), 123.45, 139.08, 149.45 (5C, pyridine-C), 143.86 (2C, 2 CH=N), 160.94, 176.55 (4C, 4C=O); MS, *m/z* (%): 582 (M^+^, 6), 584 (M^+^+2, 2), 548 (12), 546 (4), 511 (15), 435 (18), 359 (16), 204 (100), 133 (65), 105 (32), 77 (78).

*N^2^,N^6^-Bis(1-(2-(2-chloro-6-fluorobenzylidene)hydrazinyl)-1-oxopropan-2-yl)pyridine-2,6-dicarbox-amide* (**4e**). IR (KBr, cm^-1^): ν 3410-3235 (NH), 1682, 1674 (2C=O, amide); ^1^H-NMR (DMSO-d_6_): δ 1.34 (d, 6H, 2 CH_3_), 4.55 (m, 2H, 2CH), 7.24-7.72 (m, 6H, Ar-H), 8.15-8.38 (m, 5H, pyridine-H + 2CH=N), 8.80, 10.74 (2s, 4H, 4NH exchangeable with D_2_O);^ 13^C-NMR: 17.88 (2C, 2CH_3_), 51.10 (2C, 2CH), 112.68, 117.78, 124.86, 133.56, 134.48, 160.65 (12C, 2Ar-C), 124.00, 139.24, 149.56 (5C, pyridine-C), 142.94 (2C, 2 CH=N), 160.76, 176.55 (4C, 4C=O); MS, *m/z* (%): 618 (M^+^, 15), 620 (M^+^+2, 6), 488 (13), 490 (4), 359 (100), 318 (66), 275 (88), 204 (96), 133 (45), 105 (86), 77 (84).

*N^2^,N^6^-Bis(1-oxo-1-(2-(thiophen-2-ylmethylene)hydrazinyl)propan-2-yl)pyridine-2,6-dicarboxamide* (**4f**). IR (KBr, cm^-1^): ν 3390-3212 (NH), 1680, 1675 (2C=O, amide); ^1^H-NMR (DMSO-d_6_): δ 1.42 (d, 6H, 2 CH_3_), 4.46 (m, 2H, 2CH), 7.10-7.65 (m, 6H, thiophene-H), 8.22-8.35 (m, 5H, pyridine-H + 2CH=N), 8.76, 10.65 (2s, 4H, 4NH exchangeable with D_2_O);^ 13^C-NMR: 17.92 (2C, 2CH_3_), 51.08 (2C, 2CH), 126.76, 127.65, 139.32, 143.84 (8C, 2 thiophene-C), 124.05, 139.32, 149.48 (5C, pyridine-C), 132.88 (2C, 2 CH=N), 160.65, 176.62 (4C, 4C=O); MS, *m/z* (%): 525 (M^+^, 6), 442 (14), 400 (32), 329 (10), 317 (4), 196 (75), 204 (86), 133 (100), 105 (68), 77 (72).

*N^2^,N^6^-Bis(1-(2-benzylidenehydrazinyl)-2-methyl-1-oxopropan-2-yl)pyridine-2,6-dicarboxamide* (**5a**). IR (KBr, cm^-1^): ν 3360-3210 (NH), 1678, 1674 (2C=O, amide); ^1^H-NMR (DMSO-d_6_): δ 1.42 (s, 12H, 4 CH_3_), 7.25-7.82 (m, 10H, 2Ph-H), 8.12-8.25 (m, 3H, pyridine-H), 8.28 (s, 2H, 2CH=N), 8.45, 10.65 (2s, 4H, 4NH exchangeable with D_2_O); ^13^C-NMR: 25.48 (4C, 4CH_3_), 59.78 (2C, 2 NHC(CH_3_)_2_CO), 128.10, 128.90, 130.48, 133.05 (12C, 2Ph-C), 124.12, 139.01, 149.48 (5C, pyridine-C), 143.65 (2C, 2 CH=N), 160.12, 178.86 (4C, 4C=O); MS, *m/z* (%): 541 (M^+^, 6), 464 (4), 387 (12), 345 (24), 303 (100), 218 (45), 133 (65), 105 (74), 77 (48).

*N^2^,N^6^-Bis(1-(2-(4-methoxybenzylidene)hydrazinyl)-2-methyl-1-oxopropan-2-yl)pyridine-2,6-dicarbox-amide* (**5b**). IR (KBr, cm^-1^): ν 3354-3212 (NH), 1680, 1678 (2C=O, amide); ^1^H-NMR (DMSO-d_6_): δ 1.30 (s, 12H, 4 CH_3_), 3.68 (s, 6H, 2OCH_3_), 7.08 (d, 4H, Ar-H), 7.72 (d, 4H, Ar-H), 8.16 (s, 2H, 2CH=N), 8.18-8.30 (m, 3H, pyridine-H), 8.42, 10.68 (2s, 4H, 4NH exchangeable with D_2_O); ^13^C-NMR: 25.36 (4C, 4CH_3_), 59.64 (2C, 2 NHC(CH_3_)_2_CO), 54.55 (2C, 2OCH_3_), 113.98, 125.56, 129.88, 162.34 (12C, 2Ar-C), 123.48, 139.05, 149.35 (5C, pyridine-C), 143.89 (2C, 2 CH=N), 160.75, 178.85 (4C, 4C=O); MS, *m/z* (%): 602 (M^+^+1, 16), 570 (22), 539 (18), 336 (100), 218 (10), 203 (67), 205 (54), 133 (18), 118 (45), 105 (42), 77 (32).

*N^2^,N^6^-Bis(2-methyl-1-oxo-1-(2-(3,4,5-trimethoxybenzylidene)hydrazinyl)propan-2-yl)pyridine-2,6-dicarboxamide* (**5c**). IR (KBr, cm^-1^): ν 3362-3210 (NH), 1679, 1675 (2C=O, amide); ^1^H-NMR (DMSO-d_6_): δ 1.34 (s, 12H, 4 CH_3_), 3.72 (s, 18H, 6OCH_3_), 7.10 (s, 4H, Ar-H), 8.22 (s, 2H, 2CH=N), 8.18-8.28 (m, 3H, pyridine-H), 8.62, 10.48 (2s, 4H, 4NH exchangeable with D_2_O); ^13^C-NMR: 25.67 (4C, 4CH_3_), 55.82 (4C, 4OCH_3_), 59.72 (2C, 2 NHC(CH_3_)_2_CO), 59.88 (2C, 2OCH_3_), 104.05, 127.64, 141.08, 153.28 (12C, 2Ar-C), 123.75, 139.12, 149.70 (5C, pyridine-C), 145.22 (2C, CH=N), 160.98, 179.12 (4C, 4C=O); MS, *m/z* (%): 722 (M^+^+1, 14), 690 (18), 660 (6), 524 (18), 388 (12), 345 (10), 303 (100), 218 (45), 133 (56), 77 (66).

*N^2^,N^6^-Bis(1-(2-(4-chlorobenzylidene)hydrazinyl)-2-methyl-1-oxopropan-2-yl)pyridine-2,6-dicarbox-amide* (**5d**). IR (KBr, cm^-1^): ν 3360-3205 (NH), 1678, 1674 (2C=O, amide); ^1^H-NMR (DMSO-d_6_): δ 1.32 (s, 12H, 4 CH_3_), 7.44 (d, 4H, Ar-H), 7.66 (d, 4H, Ar-H), 8.22-8.37 (m, 5H, pyridine-H + 2CH=N), 8.64, 10.75 (2s, 4H, 4NH exchangeable with D_2_O); ^13^C-NMR: 26.01 (4C, 4CH_3_), 59.68 (2C, 2 NHC(CH_3_)_2_CO), 127.66, 128.88, 130.84, 135.92 (12C, 2Ar-C), 123.65, 139.10, 149.34 (5C, pyridine-C), 143.90 (2C, 2 CH=N), 160.86, 179.55 (4C, 4C=O); MS, *m/z* (%): 610 (M^+^, 10), 612 (M^+^+2, 3), 498 (18), 500 (5), 463 (6), 387 (24), 345 (35), 303 (78), 218 (100), 133 (25), 105 (30), 77 (82).

*N^2^,N^6^-Bis(1-(2-(2-chloro-6-fluorobenzylidene)hydrazinyl)-2-methyl-1-oxopropan-2-yl)pyridine-2,6-di-carboxamide* (**5e**). IR (KBr, cm^-1^): ν 3410-3232 (NH), 1680, 1676 (2C=O, amide); ^1^H-NMR (DMSO-d_6_): δ 1.35 (s, 12H, 4 CH_3_), 7.28-7.78 (m, 6H, Ar-H), 8.16-8.36 (m, 5H, pyridine-H + 2CH=N), 8.72, 10.70 (2s, 4H, 4NH exchangeable with D_2_O);^ 13^C-NMR: 26.01 (4C, 4CH_3_), 59.56 (2C, 2 NHC(CH_3_)_2_CO), 112.62, 117.66, 124.72, 133.46, 134.52, 160.60 (12C, 2Ar-C), 123.96, 139.12, 149.50 (5C, pyridine-C), 142.67 (2C, 2 CH=N), 160.66, 179.55 (4C, 4C=O); MS, *m/z* (%): 646 (M^+^, 6), 648 (M^+^+2, 2), 516 (18), 518 (6), 387 (45), 345 (100), 303 (16), 133 (75), 105 (66), 77 (80).

*N^2^,N^6^-Bis(2-methyl-1-oxo-1-((E)-2-(thiophen-2-ylmethylene)hydrazinyl)propan-2-yl)pyridine-2,6-di-carboxamide* (**5f**). IR (KBr, cm^-1^): ν 3392-3208 (NH), 1680, 1676 (2C=O, amide); ^1^H-NMR (DMSO-d_6_): δ 1.35 (s, 12H, 4 CH_3_), 7.14-7.62 (m, 6H, thiophene-H), 8.24-8.38 (m, 5H, pyridine-H + 2CH=N), 8.36, 10.15 (2s, 4H, 4NH exchangeable with D_2_O);^ 13^C-NMR: 26.02 (4C, 4CH_3_), 59.52 (2C, 2 NHC(CH_3_)_2_CO), 126.64, 127.55, 139.28, 143.92 (8C, 2 thiophene-C), 123.98, 139.36, 149.42 (5C, pyridine-C), 128.10 (2C, 2 CH=N), 160.55, 179.86 (4C, 4C=O); MS, *m/z* (%): 553 (M^+^, 16), 470 (24), 387 (13), 345 (12), 303 (24), 218 (100), 133 (860), 105 (64), 77 (54).

## 4. Conclusions

A series of pyridine-bridged 2,6-bis-carboxamide Schiff's bases was prepared starting from 2,6-pyridinedicarbonyl dichloride (**1**) and L-alanine or 2-methylalanine methyl ester. The structural assignments of the new compounds were based on chemical and spectroscopic evidence. The newly synthesized compounds **2-5** have been screened for their bactericidal and fungicidal activities, and the Schiff's bases **4b-f** and **5b-f** have significant antimicrobial activities compared to streptomycin and fusidic acid which were used as antibacterial and antifungal reference drugs, respectively. The substituted 4-methoxy- **4b,5b**, 3,4,5-trimethoxy-**4c,5c**, 4-chloro-**4d,5d**, 2-chloro-6-flouro-**4e,5e** and 2-thienyl- derivatives **4f,5f** have antimicrobial activities higher than that of **4a,5a** with an unsubstituted phenyl group.

## References

[1] Attia A., Abdel-Salam O.I., Abo-Ghalia M.H., Amr A.E. (1995). Chemical and biological reactivity of newly synthesized 2-chloro-6-ethoxy-4-acetylpyridine. Egypt. J. Chem..

[2] Attia A., Abdel-Salam O.I., Amr A.E. (1997). Utilization of 2,6-disubstituted isonicotinic acid hydrazides in the synthesis of some antibacterial agents. Egypt. J. Chem..

[3] Attia A., Abdel-Salam O.I., Amr A.E. (2000). Synthesis of some 2,6-di- and 1,2,6-trisubstituted-1,4-dihydropyridines as antimicrobial agents. Egypt. J. Chem..

[4] Amr A.E. (2000). Synthesis of some heterocyclic compounds as potential antimicrobial agents using 2,6-diacetylpyridine as synthon. Indian J. Heterocycl. Chem..

[5] Abou-Ghalia M.H., Amr A.E., Abdulla M.M. (2003). Synthesis of some new (N^α^-dipicolinoyl)-bis-L-leucyl-DL-norvalyl linear tetra and cyclic octa bridged peptides as new anti-inflammatory Agents. Z. Naturforsch..

[6] Abou-Ghalia M.H., Amr A.E. (2004). Synthesis and investigation of a new cyclo-(N^α^-dipicolinoyl) pentapeptide of a breast and CNS cytotoxic activity and an ionophoric specifity. Amino Acids.

[7] Bayrak H., Demirbas A., Karaoglu S.A., Demirbas N. (2009). Synthesis of some new 1,2,4-triazoles, their Mannich and Schiff bases and evaluation of their antimicrobial activities. Eur. J. Med. Chem..

[8] Ashok M., Holla B.S., Boojary B. (2007). Convenient one pot synthesis and antimicrobial evaluation of some new Mannich bases carrying 4-methylthiobenzyl moiety. Eur. J. Med. Chem..

[9] Karthikeyan M.S., Prasad D.J., Boojary B., Bhat K.S., Holla B.S., Kumari N.S. (2006). Synthesis and biological activity of Schiff and Mannich bases bearing 2,4-dichloro-5-fluorphenyl moiety. Bioorg. Med. Chem..

[10] Tozkoparan B., Küpeli E., Yeşilada E., Ertan M. (2007). Preparation of 5-aryl-3-alkylthio-l,2,4-triazoles and corresponding sulfones with antiinflammatory–analgesic activity. Bioorg. Med. Chem..

[11] Labanauskas L., Udrenaite E., Gaidelis P., Brukštus A. (2004). Synthesis of 5-(2,3,4-methoxy-phenyl)-4*H*-1,2,4-triazole-3-thiol derivatives exhibiting anti-inflammatory activity. II Farmaco.

[12] Navidpour L., Shafaroodi H., Abdi K., Amini M., Ghahremani M.H., Dehpour A.R., Shafiee A. (2006). Design, synthesis, and biological evaluation of substituted 3-alkylthio-4,5-diaryl-4*H*-1,2,4-triazoles as selective COX-2 inhibitors. Bioorg. Med. Chem..

[13] Maxwell J.R., Wasdahl D.A., Wolfson A.C., Stenberg V.I. (1984). Synthesis of 5-aryl-2*H*-tetrazoles, 5-aryl-2*H*-tetrazole-2-acetic acids, and [(4-phenyl-5-aryl-4*H*-1,2,4-triazol-3-yl)thio]acetic acids as possible superoxide scavengers and anti-inflammatory agents. J. Med. Chem..

[14] Amr A.E., Abo-Ghalia M., Abdalah M.M. (2006). Synthesis of novel macrocyclic peptido-calix[4]arenes and peptidopyridines as precursors for potential molecular metallacages, chemosensors and biologically active candidates. Z. Naturforsch..

[15] Amr A.E., Mohamed A.M., Ibrahim A.A. (2003). Synthesis of some new chiral tricyclic and macrocyclic pyridine derivatives as antimicrobial agents. Z. Naturforsch..

[16] Amr A.E., Mohamed A.M., Mohamed S.F., Abdel-Hafez N.A., Hammam A.G. (2006). Anticancer activities of some newly synthesized pyridine, pyrane, and pyrimidine derivatives. Bioorg. Med. Chem..

[17] Amr A.E., Sabrry N.M., Abdalla M.M., Abdel-Wahab B.F. (2009). Synthesis, antiarrhythmic and anticoagulant activities of novel thiazolo derivatives from methyl 2-(thiazol-2-ylcarbamoyl)-acetate. Eur. J. Med. Chem..

[18] Fakhr I.M., Amr A.E., Sabry N.M., Abdalah M.M. (2008). Anti-Inflammatory and analgesic activities of newly synthesized chiral peptide derivatives using (3-benzoyl-4,5-dioxo-2-phenyl-pyrrolidin-1-yl)acetic acid ethylester ass material. Arch. Pharm. Chem. Life Sci..

[19] Mohamed S.F., Flefel E.M., Amr A.E., Abd El-Shafy D.N. (2010). Anti-HSV-1 activity and mechanism of action of some new synthesized substituted pyrimidine, thiopyrimidine and thiazolopyrimidine derivatives. Eur. J. Med. Chem..

[20] Amr A.E., Ali K.A., Abdalla M.M. (2009). Cytotoxic, antioxidant activities and structure activity relationship of some newly synthesized terpenoidal oxaliplatin analogs. Eur. J. Med. Chem..

[21] Attia A., Abdel-Salam O.I., Amr A.E., Stibor I., Budesinsky M. (2000). Synthesis and antimicrobial activity of some new chiral bridged macrocyclic pyridines. Egypt. J. Chem..

[22] Abou-Zeid A.A., Shehata Y.M. (1969). A simple technique for assaying antibiotics using methylene blue as an indicator. Indian J. Pharm..

